# Fundus Autofluorescence in Lamellar Macular Holes and Pseudoholes: A Review

**DOI:** 10.1155/2019/4948212

**Published:** 2019-02-10

**Authors:** Ferdinando Bottoni

**Affiliations:** Eye Clinic, Department of Clinical Science “Luigi Sacco”, Sacco Hospital, University of Milan, Milan, Italy

## Abstract

Macular pseudoholes (MPHs) and lamellar macular holes (LMHs) have been recently defined according to spectral domain optical coherence tomography (SD-OCT) criteria. A major feature for differentiating an MPH from an LMH remains the loss of foveal tissue. The anatomy of the foveola is peculiar with the macular pigment (MP) embedded in a very thin layer of tissue underlying the internal limiting membrane and mainly constituted of a specialized group of Müller cells and Henle's fibers. Despite the near microscopic resolution (≈5–7 *μ*m) and the capability to visualize the outer retina in detail, SD-OCT may fail to ascertain whether a very small loss of this foveolar tissue has occurred. Blue-fundus autofluorescence (B-FAF) imaging is useful in this respect because even very small loss of MP can be identified, suggesting a corresponding localized loss of the innermost layers of the foveola. A definition of MP loss would help differentiating an LMH from an MPH where B-FAF imaging will be negative.

## 1. Introduction

Macular pseudoholes (MPHs) and lamellar macular holes (LMHs) have been defined in many different ways [1, 2], but the common denominator has always been whether or not a loss of foveal tissue is present. Even in the last classification by the International Vitreomacular Traction Study Group [3], MPHs are characterized by the absence of foveal tissue loss, whereas in LMHs, a partial defect of the inner fovea should be present.

Clinically, by fundus examination, both MPHs and LMHs have a similar appearance with a round and well-circumscribed reddish lesion at the center of the fovea [1, 4–11]. Functional tests, such as the Watzke-Allen test [12] and microperimetry [13], in which no scotoma is detected in either MPH and LMH, are not useful for differentiating between these conditions, and both clinical entities can lead to similarly impaired vision (median 20/40) [1–4].

Time domain (TD) [1, 7–11, 14] and spectral domain (SD) [2, 3, 15, 16] optical coherence tomography (OCT) have expanded our knowledge about pathogenesis, morphology, and progression of these clinical entities. However, despite their near-microscopic resolution (≈5–7 *μ*m) and the capability to visualize the outer retina in detail, SD-OCT may fail to ascertain whether a very small loss of foveal tissue has occurred. Blue-fundus autofluorescence (B-FAF) imaging is useful in this respect, especially in early LMHs when there is a lack of evident break in the inner fovea and dehiscence of the retina [2, 14], and the diagnosis of MPHs or LMHs remains a matter of speculation.

## 2. Pathogenesis

The pathogenesis of MPHs and LMHs is not fully understood. It has been hypothesized that an MPH is attributable to the centripetal contraction of an epiretinal membrane [4]. In contrast, an LMHs is thought to be the result of an abortive process in the formation of an FTMH. Posterior vitreous detachment is the main initiating process of the latter, but epiretinal membrane contraction has been suggested as a likely secondary factor [2, 16, 17]. This mechanism is also supported by two findings: (i) 62% to 89% of LMHs may present with an epiretinal membrane [1, 2]; and (ii) pseudo-opercula, suggestive of an aborted macular hole, have been reported in only 24% of patients with LMH [1]. Therefore, it seems that the pathogenesis of LMH cannot simply be attributed to an abortive anteroposterior traction, the classic process in FTMH formation.

## 3. Anatomy

**Figure 1:**
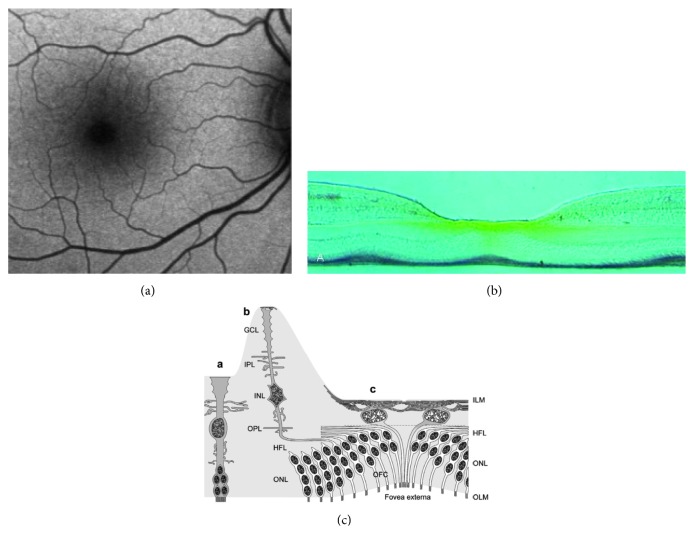
(a) Normal distribution of fundus AF. (b) Frozen section through the fovea of a rhesus monkey. Yellow macular pigment has higher concentration along Henle's fiber layer (courtesy of D. Max Snodderly, PhD, Professor of Neurobiology, College of Natural Sciences, Department of Nutritional Sciences, University of Texas, Austin, USA). (c) Reprinted from Syrbe et al. [21]. Schematic representation of the Müller cell morphology in the peripheral retina (A), foveal slope and parafovea (B), and foveola (C). The somata of Müller cells in the foveola lie in the innermost layer which is composed also by their thin inner processes forming an elaborated plait along and below the internal limiting membrane (ILM).

In a normal fundus, the distribution of fundus autofluorescence (AF) is diffuse, with decreased intensity at the optic nerve head, the retinal blood vessels, and the macula [18, 19]. Macular AF is attenuated by the presence of luteal pigment, which has a very high density in the fovea (Figure 1(a)). Originally, the foveolar macular pigment was thought to be located in the Henle fiber layer (Figure 1(b)) [20]. It has been recently demonstrated that (in addition to the typical “z-shape” Müller cells of the fovea) there are 25–35 unique Müller cells in the foveola [21]. These Müller cells are suggested to form the “Müller cell cone,” i.e., the inverted cone-like structure that overlies the area of high photoreceptor density in the primate foveola [22]. Inner processes and nuclei of these specialized Müller cells together with the axons of the highly packed foveal cones constitute the very thin layer of tissue immediately below the foveolar internal limiting membrane (ILM) (Figure 1(c)); that is, the place where the foveolar macular pigment is located at high density [21]. The density of the macular pigment decreases continuously from the center to the periphery of the foveola. The stalk of the “Müller cell cone” as well as Müller cell processes in the nerve fiber layer, inner plexiform layer (IPL), outer plexiform layer (OPL), and Henle fiber layer (HFL) in the central fovea walls also contain the macular pigment, albeit at lower density [20, 21]. Any foveal defect, including an LMH that spares the photoreceptors [2], may alter the degree of foveal AF by decreasing the amount of masking luteal pigment and thus increasing the foveal AF.

## 4. Imaging Techniques

### 4.1. Slit-Lamp Biomicroscopy

Slit-lamp biomicroscopy in patients with MPHs and LMHs may simply show the common feature of a round, reddish lesion at the center of the macula, but it is not sensitive enough to detect a small loss of foveal tissue, which is present in LMHs with preserved visual acuity. Additionally, the presence of an epiretinal membrane is not definitive in the differential diagnosis of LMH and MPH; as mentioned above, 62% to 89% of patients with LMH may have an associated epiretinal membrane [1, 2], which will always be present in MPH [4].

Haouchine et al. [1] showed that only 28% of LMH cases diagnosed with OCT were diagnosed as LMH on fundus examination. Likewise, Witkin et al. [2] reported that only 37% of LMH cases diagnosed using ultrahigh-resolution OCT were detected clinically on fundus examination. These data show the limitation of slit-lamp biomicroscopy in the diagnosis of LMH.

### 4.2. Optical Coherence Tomography

The criteria for the OCT diagnosis of MPH and LMH were originally defined by Haouchine et al. [1] and Witkin et al. [2] and subsequently confirmed by the International Vitreomacular Traction Study Group [3].

**Figure 2:**
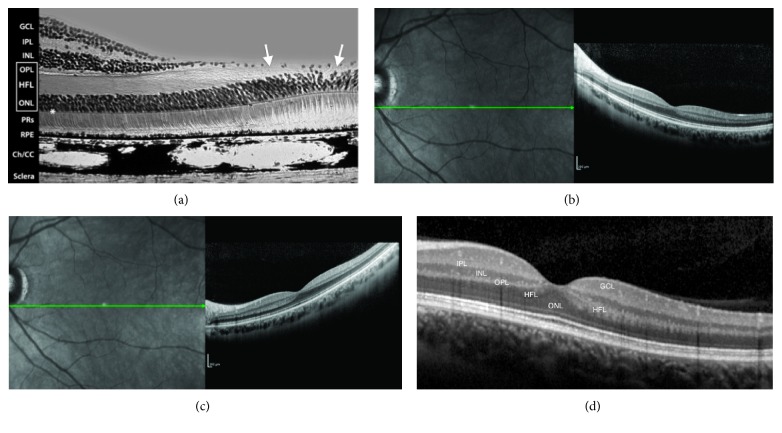
(a) Mammalian foveal histology. Henle's fiber layer continues in the foveal pit as a thin layer (arrows) (courtesy of Roger C. Wagner, PhD, Professor Emeritus of Biological Sciences, University of Delaware, Newark, Delaware, USA). (b, c) Healthy young macula. Enhanced visualization of Henle's fibers shifting the entry position of the SD-OCT beam through the pupil nasal (b) and temporal (c) to the fovea: the thin layer of fibers in the fovea remains undetected on horizontal B-scan. (d) Normal fovea. Acquired images correlate beautifully with histologic specimens (a), but the innermost layers of the retina in the foveola remain difficult to visualize in normal eyes. A less reflective layer opposite to the well-defined hyperreflective layer of Henle's fibers may be seen as continuous in the center (GCL: ganglion cell layer; IPL: inner plexiform layer; INL: inner nuclear layer; OPL: outer plexiform layer (dendritic); HFL: Henle's fiber layer (axonal OPL); ONL: outer nuclear layer).

**Figure 3:**
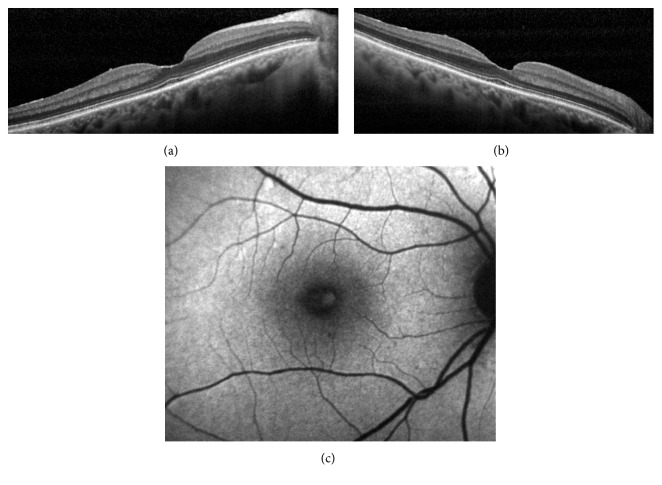
(a, b) SD-OCT revealing an irregular foveal contour without a break in the inner fovea or a dehiscence of the inner foveal retina from the outer retina. Enhanced visualization of Henle's fiber layer fails to determine whether or not a loss of tissue has occurred in the center. (c) Corresponding fundus AF image showing a lack of macular pigment. A diagnosis of LMH should be made.

The four basic criteria for the OCT diagnosis of LMH are an irregular foveal contour, a break in the inner fovea, a dehiscence of the inner foveal retina from the outer retina, and an absence of a full-thickness foveal defect with intact foveal photoreceptors. Further confusion was added to the debate by a later assertion that LMH with lamellar cleavage of their edge remain pseudoholes because there is no loss of foveal tissue as shown by SD-OCT [16]. It is clear that one of the major problems encountered in an OCT diagnosis of a foveal defect is the difficulty of determining with certainty whether there is loss of retinal tissue. Furthermore, if there is loss of foveal tissue, it is difficult to determine its anatomic location. The reason for that became apparent looking at the last SD-OCT classification of posterior ocular layers by the International Nomenclature Panel [23] who took into consideration the findings on Henle's fibers imaging [24, 25]. As already mentioned, the foveal center is made of a thick photoreceptor layer and a specialized group of Müller cells [21]; Henle's fibers are reduced to an extremely thin layer (Figure 2(a)). The entry position of the SD-OCT beam through the pupil must vary to reliably identify Henle's fibers temporal and nasal to the fovea [24, 25] (Figures 2(b) and 2(c)). The acquired images correlate beautifully with histologic specimens (Figures 2(a)–2(d)), but the inner foveolar layers remain difficult to visualize in normal eyes (Figures 2(b)–2(d)). If a LMH is suspected (i.e., loss of foveal tissue), it is even more difficult to determine whether or not this thin layer of tissue under the foveolar ILM containing the macular pigment is missing (Figures 3(a) and 3(c)).

Therefore, because of the peculiar reflectivity of the innermost retinal layers in the foveola, OCT imaging might not be sensitive enough to detect early loss of foveal tissue, that is, an initial LMH.

### 4.3. Fundus Autofluorescence

The accuracy of this technique has been reported to be comparable to that of fluorescein angiography [26, 27] for the diagnosis of FTMH.

**Figure 4:**
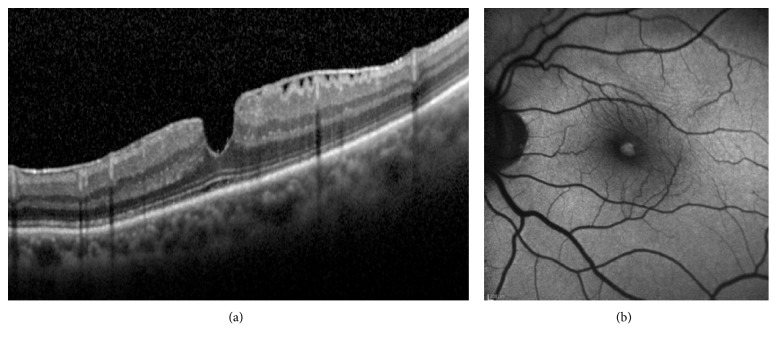
(a) SD-OCT demonstrating an epiretinal membrane, irregular foveal contour with steep edges, no break in the inner fovea or dehiscence of the inner retina from the outer retina. According to OCT criteria, a diagnosis of MPH should be established. Enhanced visualization of Henle's fiber layer fails to determine with the certainty if there is a loss of tissue in the center of the foveola. (b) Corresponding fundus AF image showing a well-defined lack of central macular pigment. A diagnosis of LMH should be made.

**Figure 5:**
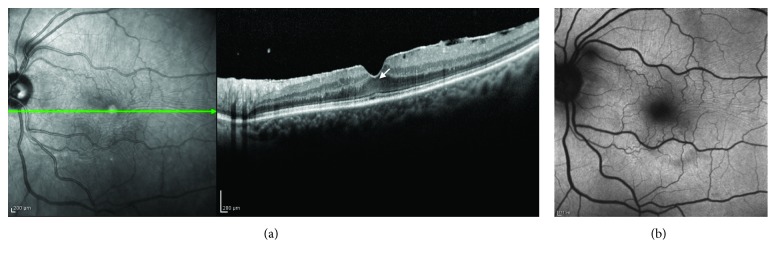
(a) Near infrared SD-OCT horizontal scan and (b) corresponding AF image in a case of epiretinal membrane with tangential tractions but no increased AF at the fovea. A thin layer of residual tissue is visible in the foveolar center (arrow). There is no lack of macular pigment.

**Figure 6:**
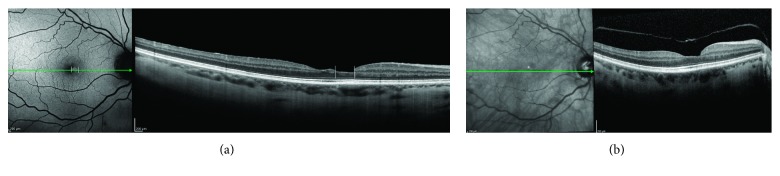
(a) B-FAF OCT image showing increased autofluorescence at the fovea (vertical lines) corresponding to the thinnest part of the foveal pit on structural OCT. (b) Same patient seven years before. A posterior vitreous detachment is visible. To be noted, remnants of foveal tissue attached at the posterior hyaloid.

Fundus autofluorescence (AF) has challenged the current OCT concepts regarding the differentiation between MPH and LMH [1–3, 5, 14, 16]. Thus, foveal lesions with SD-OCT features of MPH (i.e., irregular foveal contour with steep edges and near-normal central foveal thickness with apparent no loss of tissue) often demonstrate increased foveal AF (Figures 4(a) and 4(b)). It was already reported in a study of patients with residual retinal tissue at the bottom of the fovea classified as MPH and LMH according to the OCT profiles established by Haouchine et al. [1] that the foveal AF intensity did not differ between these two conditions [14], where increased foveal AF was found. It could be argued that increased foveal AF could be the result of dislocated macular pigment due to the tangential traction in the fovea exerted by an epiretinal membrane. There are two reasons that seem to oppose this hypothesis: first, the lack of increased AF signal in patients with idiopathic epiretinal membrane and macular pucker (Figures 5(a) and 5(b)). Second, the presence of increased foveal AF signal in eyes with an abnormal foveal contour on OCT and no signs of tangential traction in the macula (Figures 6(a) and 6(b)). Lipofuscin-laden RPE generates fundus AF [18], which in the macula is attenuated by the luteal pigment. Most of this pigment in the foveola resides below the ILM, among inner processes/nuclei of specialized Müller cells and axons originating from the foveolar cones [21]. It has been recently demonstrated that in eyes with LMHs, a strong correlation exists between the diameters of the holes measured with B-FAF and those measured at the OPL level with OCT [28]. This may indicate that indeed a loss of foveolar tissue containing the macular pigment at the OPL level in the Müller cell inverted cone is likely.

Thus, even very thin foveal defects, such as those affecting only the innermost part of the foveola sparing the photoreceptors, as in initial LMH, may increase the foveal AF. Therefore, AF findings (i.e., increased AF at the fovea) in patients with MPH and LMH as defined by SD-OCT suggest that in both conditions there could be a loss of foveal tissue.

## 5. Clinical Implications of AF Findings

The lack of a significant difference in foveal AF between LMH and MPH, as diagnosed by means of OCT imaging, raises questions concerning the validity of this OCT differentiation. If the loss of foveal tissue is considered for the diagnosis of LMH, it must be acknowledge that determining a very thin loss of tissue in the foveola may be difficult using a SD-OCT. There are two options for ascertaining such a type of loss: A direct way would imply a direct visualization of the tissue loss by OCT, with the previous limitations underlined above. An indirect way would be to determine the loss of macular pigment, which is located in the “Müller cell inverted cone” in the foveola, by means of fundus AF. An absence or decrease of the macular pigment would increase foveal AF. The anatomical localization of the macular carotenoid pigments is a complex and controversial subject with evidence suggesting that there is a pigment in both the Müller cells and the cone axons of the fovea. As Snodderly beautifully showed (Figure 1(b)) [20], the macular pigment is present not only under the foveal ILM but also deeper toward the foveal cone fibers and in the outer and inner plexiform layers. Carotenoid pigments have been demonstrated in surgically removed lamellar hole-associated epiretinal proliferation (LHEP) which is supposed to be constituted, at least in part, by Müller cells [29]. Syrbe et al. [21], more recently, could not ascertain with certainty the exact location of the macular pigment if not confirming that it is inside the Müller cell inverted cone. That is an important clue for diagnosing tissue loss in the fovea because an increased B-FAF in the fovea would imply the absence of a masking element that should be normally there.

Whether or not this can be used for establishing a differential diagnosis between LMH and MPH remains to be further investigated. However, given the uncertainty encountered in the direct diagnosis of foveal tissue loss with OCT, B-FAF imaging may be of help as to this purpose.

An accurate diagnosis of FTMH, LMH, and MPH is important to determine the proper surgical treatment of these lesions. Different options may be selected according to the OCT and fundus AF imaging findings. For instance, in the absence of foveal AF, the integrity of the foveal tissue is almost certainly confirmed. Therefore, it is likely that removal of the epiretinal membrane alone is all that is needed in such cases. However, if foveal AF is present, a loss of foveal tissue has very likely occurred, and the decision to operate will depend on many factors, such as the residual visual acuity and progression of signs and symptoms.

## Additional Points

Fundus AF imaging is becoming increasingly important in the examination of many macular diseases, including LMH and MPH. In both, a similar degree of functional loss may occur. In such cases, an increased AF signal at the fovea signifies a loss of foveal tissue that is the macular pigment.
